# Prognostic value of miR-221 in human malignancy: evidence from 3041 subjects

**DOI:** 10.1186/s12885-019-6079-1

**Published:** 2019-08-30

**Authors:** Kangkang Liu, Lining Wang, Erlin Sun

**Affiliations:** Department of Urology, Tianjin institute of urology, The 2nd Hospital of Tianjin Medical University, No 23, PingJiang Road, Hexi District, Tianjin, 300211 People’s Republic of China

**Keywords:** MiR-221, Human carcinoma, Prognosis, Meta-analysis

## Abstract

**Background:**

MiR-221, acting as onco-miR or oncosuppressor-miR, plays an important role in tumor progression; however, the prognostic value of miR-221 in human carcinomas is controversial and inconclusive. The objective of our study was to conducted a systematic review and meta-analysis of miR-221 in various types of human cancers.

**Methods:**

An online search of up-to-date electronic databases, including PubMed and Embase, was conducted to identify as many relevant papers as possible. 32 papers involving 3041 patients with different carcinomas were included in the analysis. Hazard ratios (HRs) of miR-221 were used to evaluate prognostic values.

**Results:**

Thirty-two papers involving 15 cancers were included. MiR-221 was associated with a worse overall survival (OS) in patients, and a combined HR was 1.93 (95% CI of 1.43–2.60, 2080 patients, 22 studies, I-squared = 80.4%, *P = 0.000*); however, the combined HR for relapse-free survival (RFS) was 1.37 (95% CI of 0.75–2.48, 625 patients, 7 studies, I-squared = 78.8%, *P = 0.000*), and disease-free survival (DFS) was 1.24 (95% CI of 0.60–2.56, 539 patients, 5 studies, I-squared = 81.8%, *P = 0.000*).

**Conclusion:**

MiR-221 was shown to be associated with a poor OS in human carcinomas, and thus may serve as a useful predictor of clinical outcomes.

**Electronic supplementary material:**

The online version of this article (10.1186/s12885-019-6079-1) contains supplementary material, which is available to authorized users.

## Background

MicroRNAs (miRNAs), small noncoding single-stranded RNAs, play a pivotal role in diverse cellular processes through post-transcriptional regulation of gene expression [1]. MiRNAs are now known to play an essential role in malignancy, functioning as tumor suppressors and oncogenes [2]. The expression of miRNAs is abnormal in different carcinomas and miRNAs are involved in the development and progression of disease [3]. As favorable or unfavorable prognostic biomarkers, many miRNAs are associated with patients’ survival in different cancers [4–6].

MiR-221, located on human chromosome X, is upregulated in many different cancers. As an onco or oncosuppressor-miR, miR-221 plays an important role in tumor progression [7]. High expression of miR-221 is associated with a worse survival in patients with different cancers, such as liver, laryngeal, and lung cancers [8–10]. It has also been reported that miR-221 is a favorable factor in predicting the prognosis of ovarian and renal cancers [11, 12]. Because of the controversy involving the association between miR-221 and survival among patients with different carcinomas, we conducted a systematic review and meta-analysis of the prognostic value of miR-221 in human cancers.

## Methods

### Search strategy

The objective of our study was to summarize the prognostic value of miR-221 in human carcinomas. We conducted an online search of up-to-date electronic databases, including PubMed and Embase, to identify as many relevant papers as possible. The search was performed by professional literature librarian. The key words, “miR-221” and “cancer” were used. The details of the search strategy in two databases were shown (Additional file 6: Table S1). The reference lists of review papers were hand-searched for further relevant studies. The last search update was performed on July 20, 2019.

### Risk of bias assessment

We systematically assessed the quality of all included studies according to the guidelines that we previously described [13]. The assessment details of the studies are as follows: 1) clear information about populations and nations of included participants; 2) clear information about the type of carcinomas involved; 3) clear information about study design (prospective or retrospective); 4) clear information about the arrays used to measure the expression of miR-221; 5) clear information about the type of outcome assessment; and 6) clear information about follow-up. Studies that met the above criteria were included.

The evaluation process was performed by two independent authors, all HRs were extracted directly from papers. The risk of bias was assessed by Cochrane B or Newcastle-Ottawa Scale (NOS).

### Statistical analysis

All statistical analyses were conducted using Stata12.0. The HRs with corresponding 95% CIs were used to estimate the strength of the relationship between miR-221 and prognosis. The results were displayed by forest plots. The heterogeneity assumption of the pooled HR was verified by a χ^2^-based Cochran Q test and Higgins I^2^ statistic, with an I-squared> 50% and/or a *P* < 0.1 indicating heterogeneity. Considering the heterogeneity of different articles, a random effect was performed in this meta-analysis. Potential publication bias was determined by Begg’s test with a funnel plot.

## Results

### Characters of included studies

**Fig. 1:**
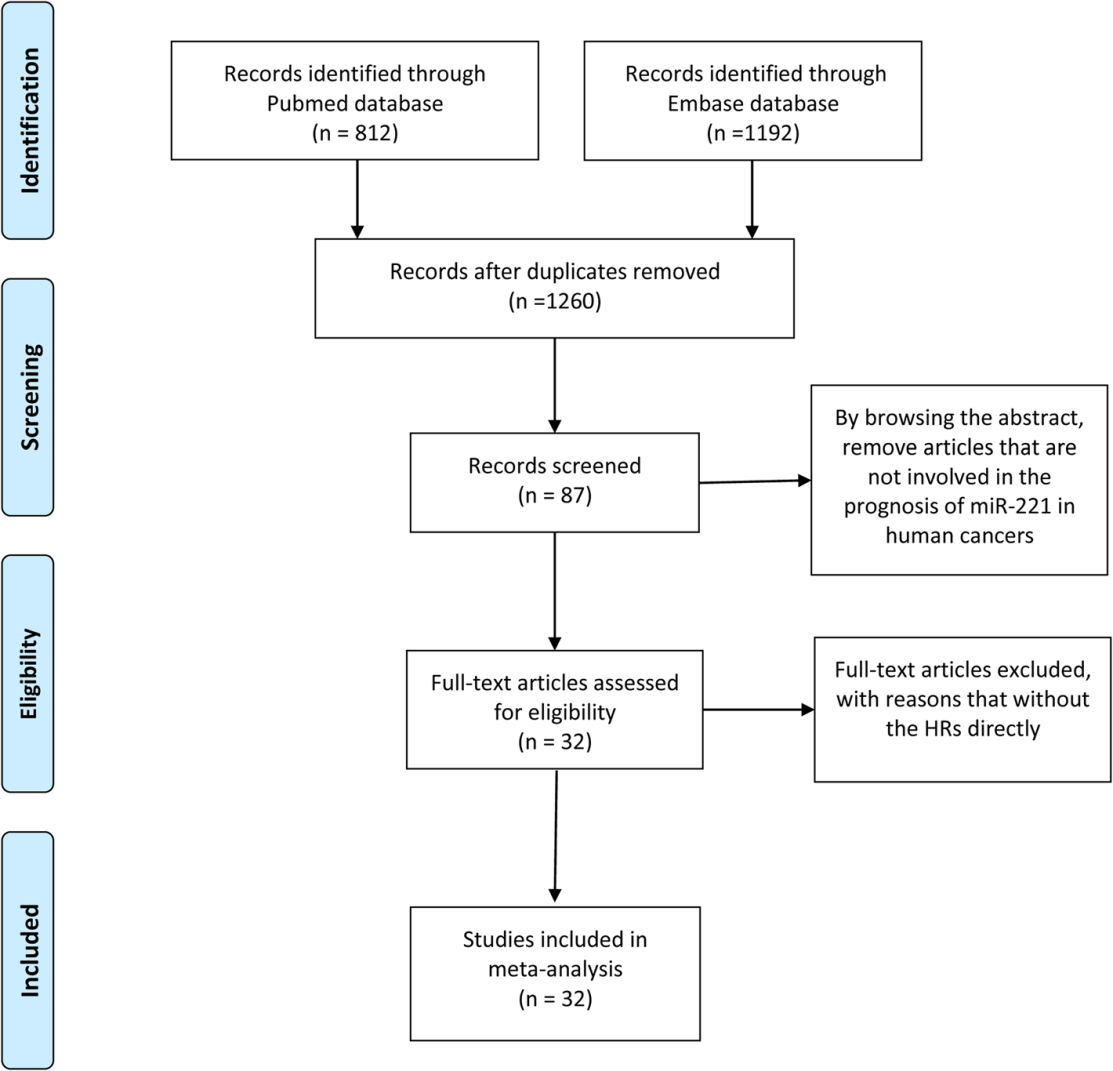
Flow chart of study selection process

812 and 1192 papers were identified from the databases of Pubmed and Embase respectively. By browsing the titles and abstracts, the articles that were duplicates or not involved in the prognostic value of miR-221 in human cancers were excluded. Then, according to full-text assessment, 32 papers researching the prognostic value of miR-221 in human carcinomas with sufficient data were included in our study (Fig. 1). All included papers have relatively high-quality assessment of the risk of bias, which met the requirements for inclusion.

A total of 3041 patients with different cancers were involved, including ovarian cancer [11, 14], bladder cancer [15], osteosarcoma [16, 17], liver cancer [8, 18–22], laryngeal cancer [9], thyroid cancer [23], lung cancer [10, 24], gastric-colon cancer [25–28], renal cancer [12], breast cancer [29–32], prostate cancer [33–36], cutaneous malignant melanoma (CMM) [37], acute lymphoid leukemia (ALL) [38, 39], and NK/T-cell lymphoma [40]. 20 of them were conducted in Asia (18 in China and 2 in Korea), the remaining studies were conducted in other countries, including Greece, the USA, Egypt, Germany, Italy, and Brazil. The origins of miR-221 in most studies were derived from tumor tissues; 7 were from serum/plasma and 2 were from bone marrow. Quantitative real time PCR (qRT-PCR) was performed in most of the studies for quantification of miR-221; two studies used in-situ hybridization (ISH) to detect miR-221 expression. The details of these papers were summarized in Table 1.

**Table 1:** Characters of included 32 papers about the prognostic value of miR-221 in human carcinomas

Study	Nation	Patients	cancer	Survival	HR(95% CI)	*P* value	Origins	Measure	Cutoff	Analysis
Wu Q (2017)	China	74	Ovarian cancer	OS	0.395 (0.196–0.796)	0.009	Tumor	qRT-PCR	Median	Univariate
Tsikrika (2017)	Greece	159	Bladder cancer	DFS PFS	0.712(0.380–1.335) 1.396(0.539–3.620)	0.290 0.492	Tumor	qRT-PCR	ROC	Univariate
Deng (2017)	China	125	Breast cancer	DFS	0.480 (0.263–0.879)	0.017	Tumor	qRT-PCR	Median	Multivariate
Nakka (2017)	USA	32	Osteosarcoma	OS	0.733(0.486–1.1055)	0.139	Tumor	qRT-PCR/ISH	Quartation	Multivariate
Xie D (2017)	China	70	Live cancer	OS	1.743 (1.004–3.772)	0.012	Tumor	qRT-PCR	Median	Multivariate
Hussein (2017)	Egypt	50	Laryngeal cancer	OS	6.5 (1.8–22.5)	0.003	Tumor	qRT-PCR	ROC	Univariate
Dai L (2017)	China	78	Thyroid cancer	RFS	1.41(1.14–1.95)	0.007	Tumor	qRT-PCR	Median	Multivariate
Chen F (2017)	China	135	HCC	DFS OS	2.846 (1.564–5.181) 2.969 (1.629–5.408)	0.001 < 0.001	Tumor	qRT-PCR	Median	Multivariate
Zhang Y (2016)	China	104	Lung cancer	OS	1.873(1.267–2.768)	0.002	Tumor	qRT-PCR	Median	Multivariate
Yang Z (2015)	China	108	Osteosarcoma	OS RFS	7.66(1.83–15.92) 6.82(1.33–13.69)	0.01 0.01	Serum	qRT-PCR	Median	Multivariate
Cai K (2015)	China	182	Colon cancer	OS	2.394 (1.210–4.910)	0.006	Tumor	qRT-PCR	Median	Multivariate
Tao K (2014)	China	90	Colon cancer	OS	2.043 (1.095–3.812)	0.025	Tumor	qRT-PCR	Median	Multivariate
Vergho (2014)	Germany	74	Renal cancer	CSS	0.47 (0.22–1.00)	0.0527	Tumor	qRT-PCR	ROC	Multivariate
Lv J (2014)	China	117	Lung cancer	OS	2.425 (1.314–4.475)	0.005	Tumor	qRT-PCR	Median	Multivariate
Li P (2014)	China	72	CMM	OS DFS	3.189(1.782–6.777) 2.119(1.962–8.552)	0.007 0.01	Serum	qRT-PCR	Median	Multivariate
Gyongyosi B (2014)	Italy	20	HCC	OS PFS	1.92(0.61–6.10) 1.32(0.47–3.66)	0.29 0.58	Tumor	qRT-PCR	Median	Univariate
Falkenberg (2013)	Germany	86	Breast cancer	MFS	2.57(1.1073–5.9647)	0.028	Tumor	qRT-PCR	ROC	Multivariate
Hong (2013)	China	96	Ovarian cancer	OS	2.243(1.1357–4.4300)	0.020	Serum	qRT-PCR	Mean	Multivariate
Gimenes (2013)	Brazil	48	ALL	OS DFS	2.31(0.92–5.81) 1.54 (0.57–4.17)	0.074 0.391	Marrow	qRT-PCR	Median	Multivariate
Karakatsanis (2013)	Greece	60	HCC	OS	1.72 (1.32–2.50)	0.002	Tumor	qRT-PCR	Mean	Multivariate
Amankwah (2013)	USA	65	Prostate cancer	RFS	1.79 (0.67–4.76)	0.25	Tumor	qRT-PCR	Median	Multivariate
Liu K (2012)	China	92	Gastric cancer	OS	2.322 (1.1116–4.8505)	0.025	Tumor	qRT-PCR	Mean	Multivariate
Hanna (2012)	USA	377	Breast cancer	OS	0.70 (0.51–0.97)	0.0312	Tumor	ISH	Quartation	Multivariate
Kang (2012)	Korea	92	Prostate cancer	RFS	0.360 (0.171–1.896)	0.570	Tumor	qRT-PCR	Median	Univariate
Li J (2011)	China	46	HCC	OS	1.903(1.235–2.981)	0.018	Serum	qRT-PCR	Mean	Multivariate
Yoon (2011)	Korea	115	HCC	RFS	3.07 (1.56–6.07)	0.001	Tumor	qRT-PCR	Mean	Multivariate
Zhao R (2011)	China	93	Breast cancer	OS	6.871 (1.967–23.997)	0.003	plasma	qRT-PCR	Median	Multivariate
Schaefer (2010)	Germany	75	Prostate cancer	RFS	0.93 (0.3–2.89)	0.902	Tumor	qRT-PCR	Median	Univariate
Wang (2010)	China	32	ALL	OS	0.538(0.30–0.9648)	0.038	Marrow	qRT-PCR	Median	Multivariate
Spahn (2010)	Germany	92	Prostate cancer	RFS	0.525(0.29–0.95)	0.032	Tumor	qRT-PCR	ROC	Multivariate
Pu (2010)	China	103	Colon cancer	OS	3.478(1.038–11.654)	0.043	Plasma	qRT-PCR	Youden	Multivariate
Guo (2010)	China	79	Lymphoma	OS	5.714(1.782–18.18)	0.003	Plasma	qRT-PCR	Youden	Multivariate

Note: *HCC* Hepatocellular carcinoma; *ALL* Acute lymphoid leukemia; *CMM* Cutaneous malignant melanoma; *ROC* Receiver operating characteristic curve; Youden, Youden index; *OS* Overall survival; *RFS* Relapse-free survival; *DFS* Disease-free survival; *PFS* Progression-free survival; *CSS* Cancer-special survival; *MFS* Metastasis-free survival; *qRT-PCR* Quantitative Real Time PCR; *ISH* In-situ hybridization

### Association of miR-221 with overall survival (OS)

**Fig. 2:**
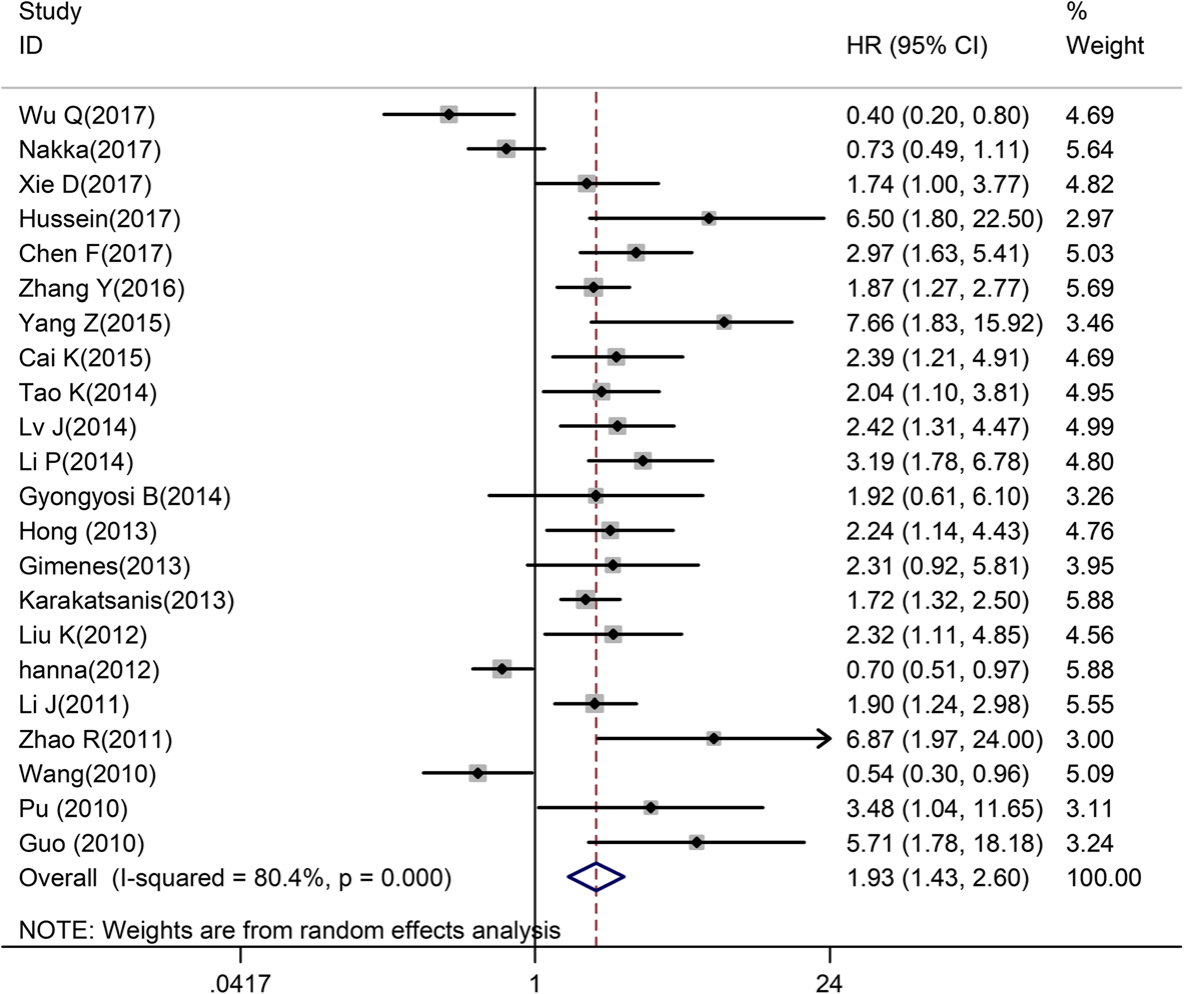
Forrest plots of the studies that evaluated the hazard ratios (HRs) of high miR-221 expression as compared to low expression in OS

A total of 22 articles researched the association between miR-221 and OS among different carcinomas. Generally, miR-221 was associated with a poor OS, with a pooled HR was 1.93 (95% CI of 1.43–2.60, 2080 patients, 22 studies, I-squared = 80.4%, *P = 0.000*) (Fig. 2). Due to the heterogeneity, subgroup analyses were performed. Most of the studies originated from China, thus we divided the patients into Asian (Chinese) and non-Asian groups. MiR-221 was significantly related to the OS of Chinese patients (HR = 2.14 (1.53–2.99), 1493 patients, 16 studies, I-squared = 74.2%, *P = 0.000*), but not non-Asian patients (HR = 1.44 (0.83–2.47), 587 patients, 6 studies, I-squared = 83.3%, *P = 0.000*) (Table 2 and Additional file 1: Figure S1). Then, we divided studies according to the number of included individuals. The combined HR was 2.28 (95% CI of 1.29–4.41, 1126 patients, 7 studies, I-squared = 85.9%, *P = 0.000*) in studies with more than 100 participants, and the HR was 1.80 (95% CI of 1.25–2.59, 954 patients, 15 studies, I-squared = 78.4%, *P = 0.000*) in studies with less than 100 patients (Table 2 and Additional file 2: Figure S2); however, heterogeneity still existed in these subgroups.

**Table 2:** Subgroup analysis of association between the expression of miR-221 and OS

Categories	Subgroups	No of studies	Pool HR	95% CI	Result	I^2^	P
All	OS	22	1.93	1.43–2.60	S	80.4%	0.000
Countries	Asian (Chinese)	16	2.14	1.53–2.99	S	74.2%	0.000
	Non-Asian	6	1.44	0.83–2.47	NS	83.3%	0.000
Samples origins	Tumor tissues	13	1.61	1.13–2.29	S	81.4%	0.000
	Serum/blood	7	3.25	2.15–4.92	S	42.2%	0.109
	Marrow	2	1.07	0.26–4.43	NS	85.4%	0.009
Sample sizes	> 100	7	2.28	1.29–4.11	S	85.9%	0.000
	≤100	15	1.80	1.25–2.59	S	78.4%	0.000
Cancer types	Colon cancer	4	2.33	1.60–3.38	S	0.0%	0.897
	Liver cancer	5	1.91	1.53–2.38	S	0.0%	0.633
	Lung cancer	2	2.02	1.45–2.81	S	0.0%	0.486
	ALL	2	1.07	0.26–4.43	NS	85.4%	0.009
	Breast cancer	2	2.02	0.22–18.81	NS	91.7%	0.001
	Osteosarcoma	2	2.24	0.23–22.29	NS	93.7%	0.000
	Ovarian cancer	2	0.94	0.17–5.17	NS	91.8%	0.000

Note: *ALL* Acute lymphoid leukemia; *S* Significant; *NS* Non-significant

We further analyzed the subgroups divided based on different cancers. The cancers investigated in more than one paper were included. The results showed that the HR was 2.33 (95% CI of 1.60–3.38, 467 patients, 4 studies, I-squared = 0.0%, *P = 0.897*) in colon cancer, 1.91 (95% CI of 1.53–2.38, 331patients, 5 studies, I-squared = 0.0%, *P = 0.633*) in liver cancer, and 2.02 (95% CI of 1.45–2.81, 221 patients, 2 studies, I-squared = 0.0%, *P = 0.486*) in lung cancer (Table 2 and Additional file 3: Fig. S3). However, the combined HR was 1.07 (95% CI of 0.26–4.43, 80 patients, 2 studies, I-squared = 85.4%, *P = 0.009*) in ALL, 2.02 (95% CI of 0.22–18.81, 470 patients, 2 studies, I-squared = 91.7%, *P = 0.001*) in breast cancer, 2.24(95% CI of 0.23–22.29, 140 patients, 2 studies, I-squared = 93.7%, *P = 0.000*) in osteosarcoma and 0.94 (95% CI of 0.17–5.17, 170 patients, 2 studies, I-squared = 91.8%, *P = 0.000*) in ovarian cancer (Table 2 and Additional file 4: Figure S4).

Interestingly, the origins of miR-221 in 7 of the papers were derived from serum or plasma. The combined HR was 3.25 (95% CI of 2.15–4.92, 597 patients, 7 studies, I-squared = 42.2%, *P = 0.109*), which suggested that the expression of miR-221 in serum/plasma was associated with a worse OS. The results differed from a previous study [41]. In addition, the combined HR of the studies from tumor tissues was 1.61 (95% CI of 1.13–2.29, 1403 patients, 13 studies, I-squared = 81.4%, *P = 0.000*), and the combined HR from marrow was 1.07 (95% CI of 0.26–4.43, 80 patients, 2 studies, I-squared = 85.4%, *P = 0.009*) (Table 2 and Additional file 5: Figure S5).

### Association of miR-221 with relapse-free survival (RFS)/ disease-free survival (DFS)

**Fig. 3:**
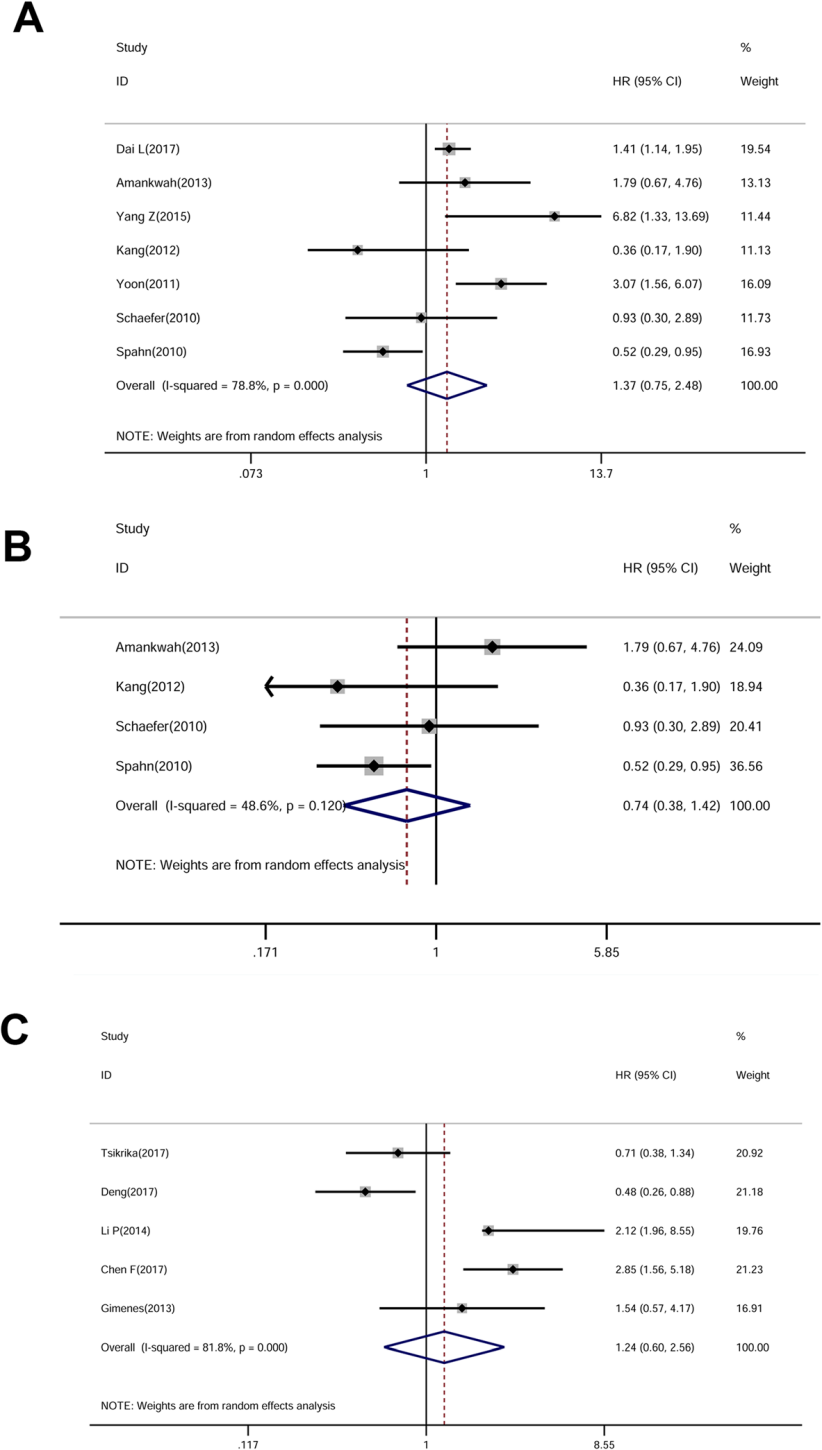
Forrest plots of the studies that evaluated the HRs of high miR-221 expression as compared to low expression in RFS/DFS (**a**) RFS (**b**) RFS of prostate cancer (**c**) DFS

Seven of papers reported an association between miR-221 and RFS. The combined HR was 1.37 (95% CI of 0.75–2.48, 625 patients, 7 studies, I-squared = 78.8%, *P = 0.000*) (Fig. 3a). Notably, 4 of them focused on prostate cancer. The combined HR was 0.74 (95% CI of 0.38–1.42, 324 patients, 4 studies, I-squared = 48.6%, *P = 0.120*), which demonstrated that miR-221 tended to be a favorable predictor of RFS in prostate cancer patients (Fig. 3b). Besides, 5 of studies focused on the DFS of patients, the combined HR was 1.24 (95% CI of 0.60–2.56, 539 patients, 5 studies, I-squared = 81.8%, *P = 0.000*) (Fig. 3c). Due to the limited number of papers mentioned about RFS/DFS of patients, we were not able to analyze the causes of heterogeneity.

### Publication bias

**Fig. 4:**
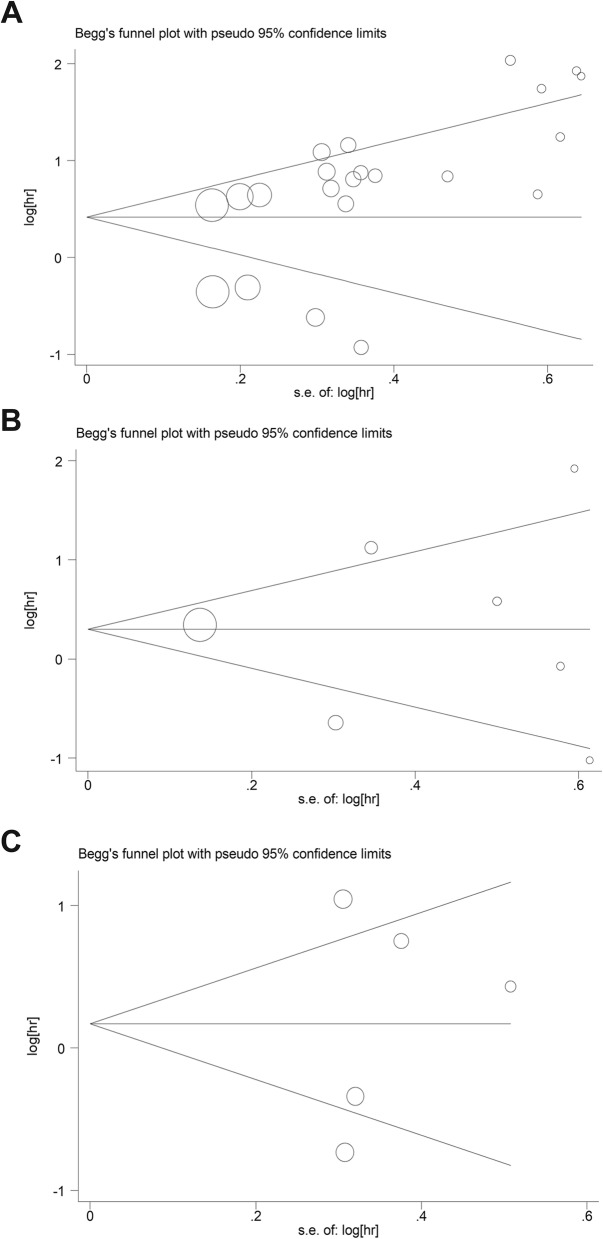
Funnel plots of Begg’s test included in the meta-analysis of (**a**) OS (**b**) RFS (**c**) DFS

Both Begg’s and Egger’s tests were performed to estimate the potential publication bias in our study. A *P < 0.05* indicated the existence of publication bias. There was no apparent publication bias in papers with respect to RFS and DFS; however, we evaluated the potential publication bias with respect to OS (*P = 0.015*). The funnel plots of Begg’s test were shown in Fig. 4.

## Discussion

MicroRNAs have been reported to be aberrantly expressed and play an important role in predicting the prognosis of human carcinomas [42]. It has been recommended to classify miRNAs into two categories (oncogenes and onco-suppressor miRNAs), which regulate tumor oncogenes or suppressor genes, respectively [43]. MiR-221, either as an oncogene or tumor suppressor gene, is involved in tumor progression in many different cancers. MiR-221 promotes the tumor progression of cancers, such as bladder, prostate, and breast cancers, by targeting downstream molecules, including PTEN, E-cadherin, and suppressors of cytokine signaling 1, 3 (SOCS1, 3) [44–48]. In contrast, miR-221 inhibits tumor progression in diseases such as pancreatic and ovarian cancers, by targeting factors, such as SOCS3 and ADP-ribosylation factor-4 (ARF4) [11, 49]. Interestingly, the dual role of miR-221 has been found in some cancers, such as pancreatic cancer [49–51]. It has been reported that some miRNAs (miR-21 and miR-155) are related to unfavorable clinical outcomes of pancreatic cancer [52]; however, there is still a lack of studies on the prognostic value of miR-221, which will be worth further exploration. Taken together, miR-221 has a dual role in tumor progression of different cancers. Future studies are necessary to elucidate the mechanisms underlying miR-221 in oncology.

The prognostic values of miR-221 have been investigated in different kinds of cancers; however, the roles of miR-221 in different studies have been controversial and inconclusive. A meta-analysis involving miR-221 in human cancers was conducted by Yang et al.in 2014 [53]. Subsequently, additional studies researching the prognostic value of miR-221 have been published in recent years and the results of those studies are inconsistent. Thus, an updated systematic review and meta-analysis was necessary to ascertain the prognostic value of miR-221.

Recently, Zhang et al. conducted a review and meta-analysis of the prognostic value of miR-221/miR-222 in human malignancy [54]; however, we noted that many relevant articles were omitted and some of the results should be re-summarized. Therefore, in the current study we systematically summarized the prognostic value of miR-221 according to the papers published in recent years. The results showed that miR-221 was associated with a worse OS of various of cancers. In agreement with our results, miR-222 (miR-221 highly homologous miRNA) was reported to be associated with poor survival [55]. Subgroup analysis showed that miR-221 was related to the OS of Chinese, but not non-Asians. Regarding different cancers, we found that miR-221 was significantly related to the OS of colon, liver, and lung cancers, but not associated with ALL, osteosarcoma, breast cancer, and ovarian cancer. With respect to the methods of miR-221 quantification, because there were limited articles using the ISH method, subgroup analysis using methods of miR-221 quantification did not produce meaningful results. In addition, the relationship between miR-221 and RFS/DFS of patients was indefinite, but high expression of miR-221 tended to be associated with a favorable RFS in prostate cancer patients. With the limited number of relevant papers, more studies are needed to confirm these conclusions.

Interestingly, Rong et al. reported that the association between the expression of miR-221 in serum/plasm and prognosis of patients was non-significant (HR = 0.94 (0.47–1.87), I-squared = 84.2%, *P = 0.000*) [41]; however, in our study, 7 papers focusing on miR-221 from serum/plasma were included. We found that miR-221 was related to a poor OS. The inconsistency of the two studies was possibly due to the different standards used in the articles, and more published studies were added in our study. The *Guo* et al. study was included in our study and *Rong* et al. study [40]. In the *Guo’s* study, the HR of multivariate analysis was calculated using high expression of miR-221 as a baseline. Therefore, the HRs directly obtained from this study should be transformed.

There were limitations in the current study that must be mentioned. First, the numbers of studies with some cancers were limited and it was difficult to conclude that a reliable association existed between miR-221 with those cancers. More studies researching miR-221 in different cancers will be necessary in the future. Second, although subgroup analysis was performed, the heterogeneity still existed in some groups. With the limited number of papers, we could not adequately explore the reasons for heterogeneity. Third, studies with negative results were generally less likely to be published. Therefore, we could not deny the potential existence of publication bias.

## Conclusion

By summarizing the results of published papers about the prognostic value of miR-221 in human cancer, we found that high expression of miR-221 was associated with a worse OS; however, the association of miR-221 with RFS/DFS was not significant. Future studies with a larger number of cases are recommended to validate the role of miR-221 in human carcinomas.

## Additional files

Additional file 1: **Figure S1.** Subgroup analyses of the studies that evaluated the HRs of high miR-221 expression as compared to low expression in OS by nations, (A) Chinses (B) non-Asian (TIF 24678 kb)

Additional file 2: **Figure S2.** Subgroup analyses of the studies that evaluated the HRs of high miR-221 expression as compared to low expression in OS by the number of individuals, (A) > 100 (B) ≤100 (TIF 24678 kb)

Additional file 3: **Figure S3.** Subgroup analyses of the studies that evaluated the HRs of high miR-221 expression as compared to low expression in OS by cancers, (A) Colon cancer (B) Live cancer (C) Lung cancer (TIF 15382 kb)

Additional file 4: **Figure S4.** Subgroup analyses of the studies that evaluated the HRs of high miR-221 expression as compared to low expression in OS by cancers, (A) ALL (B) Breast cancer (C) Osteosarcoma (D) Ovarian cancer (DOCX 19 kb) (TIF 14956 kb)

Additional file 5: **Figure S5.** Subgroup analyses of the studies that evaluated the HRs of high miR-221 expression as compared to low expression in OS by origins, (A) Tumor tissues (B) Serum/plasm (C) Marrow (TIF 24676 kb)

Additional file 6: **Table S1.** Literature search strategy of PubMed database. The Embase database is searched in a similar way to PubMed. (DOCX 19 kb)

## Data Availability

The datasets analyzed during the current study are available in the PubMed and Embase repositories. Persistent web links of each study included in the datasets are provided in the “Reference” part.

## Abbreviations

ALL: Acute lymphoid leukemia
CI: Confidence interval
CMM: Cutaneous malignant melanoma
DFS: Disease-free survival
HR: Hazard ratio
OS: Overall survival
RFS: Relapse-free survival
