# Supplementary figures and images for: Prognostic value of miR-221 in human malignancy: evidence from 3041 subjects
Source: BMC Cancer. 2019 Aug 30;19:867. doi: 10.1186/s12885-019-6079-1 (PMC6717359; doi:10.1186/s12885-019-6079-1)

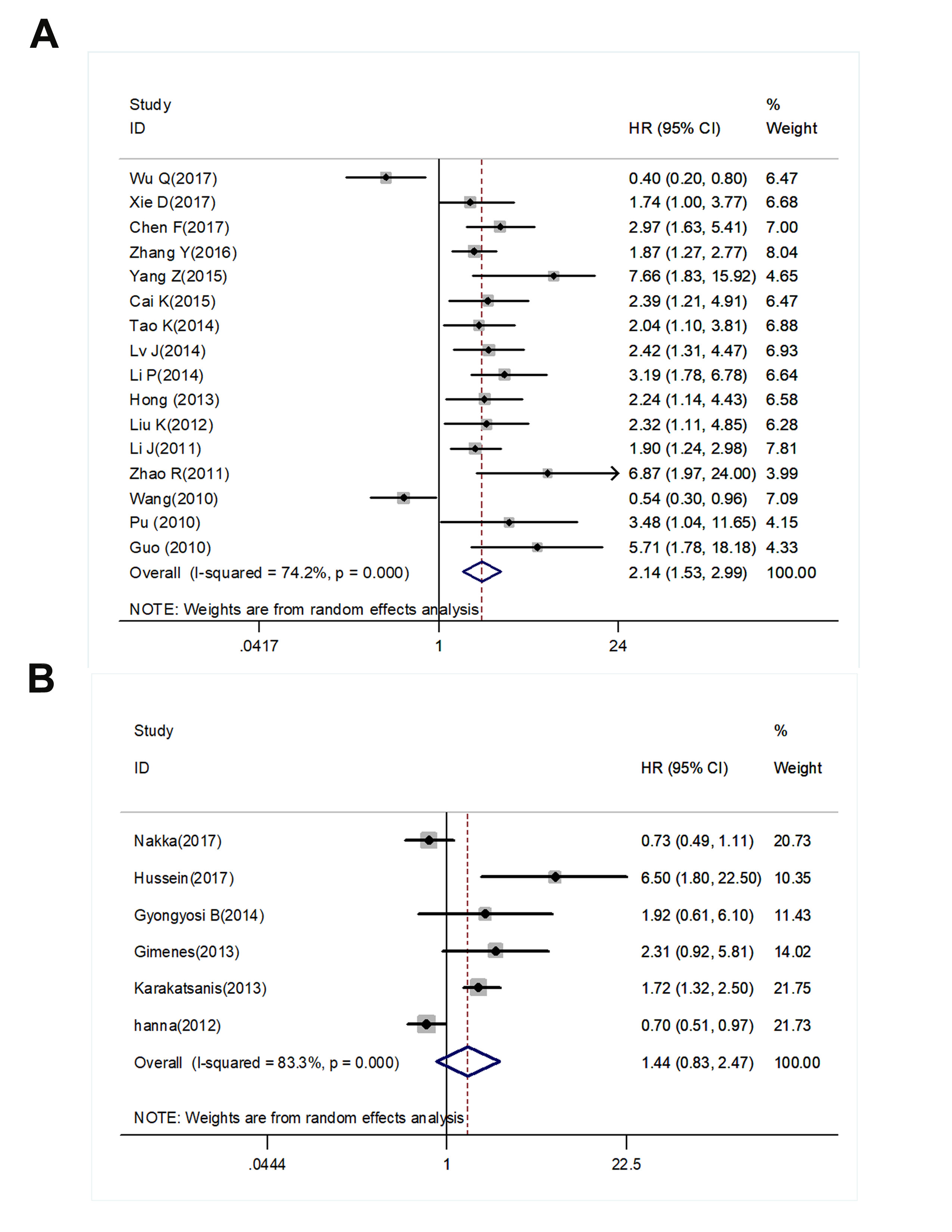

Supplement: Supplementary file 1 — Figure S1. Subgroup analyses of the studies that evaluated the HRs of high miR-221 expression as compared to low expression in OS by nations, (A) Chinses (B) non-Asian (TIF 24678 kb) [file 12885_2019_6079_MOESM1_ESM.tif]

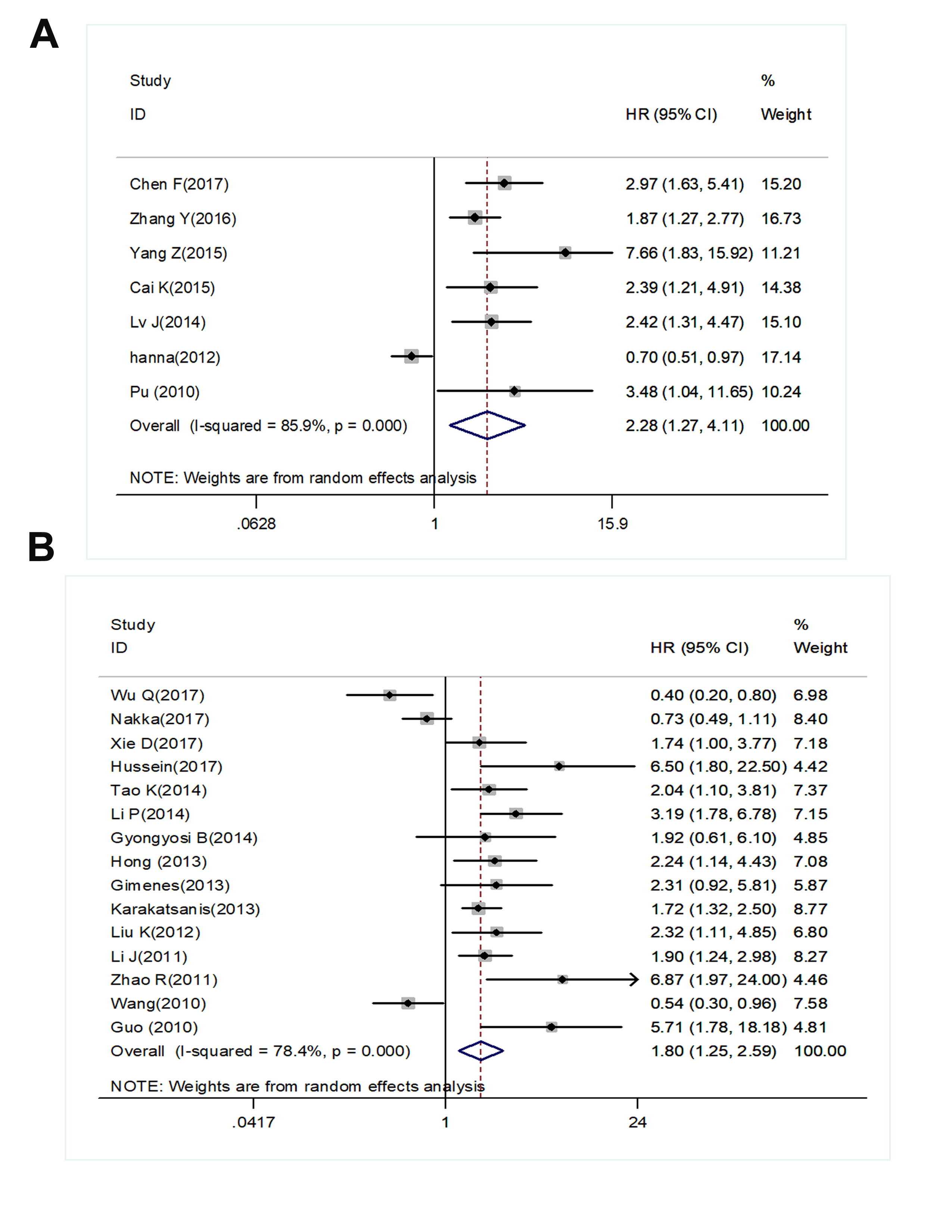

Supplement: Supplementary file 2 — Figure S2. Subgroup analyses of the studies that evaluated the HRs of high miR-221 expression as compared to low expression in OS by the number of individuals, (A) > 100 (B) ≤100 (TIF 24678 kb) [file 12885_2019_6079_MOESM2_ESM.tif]

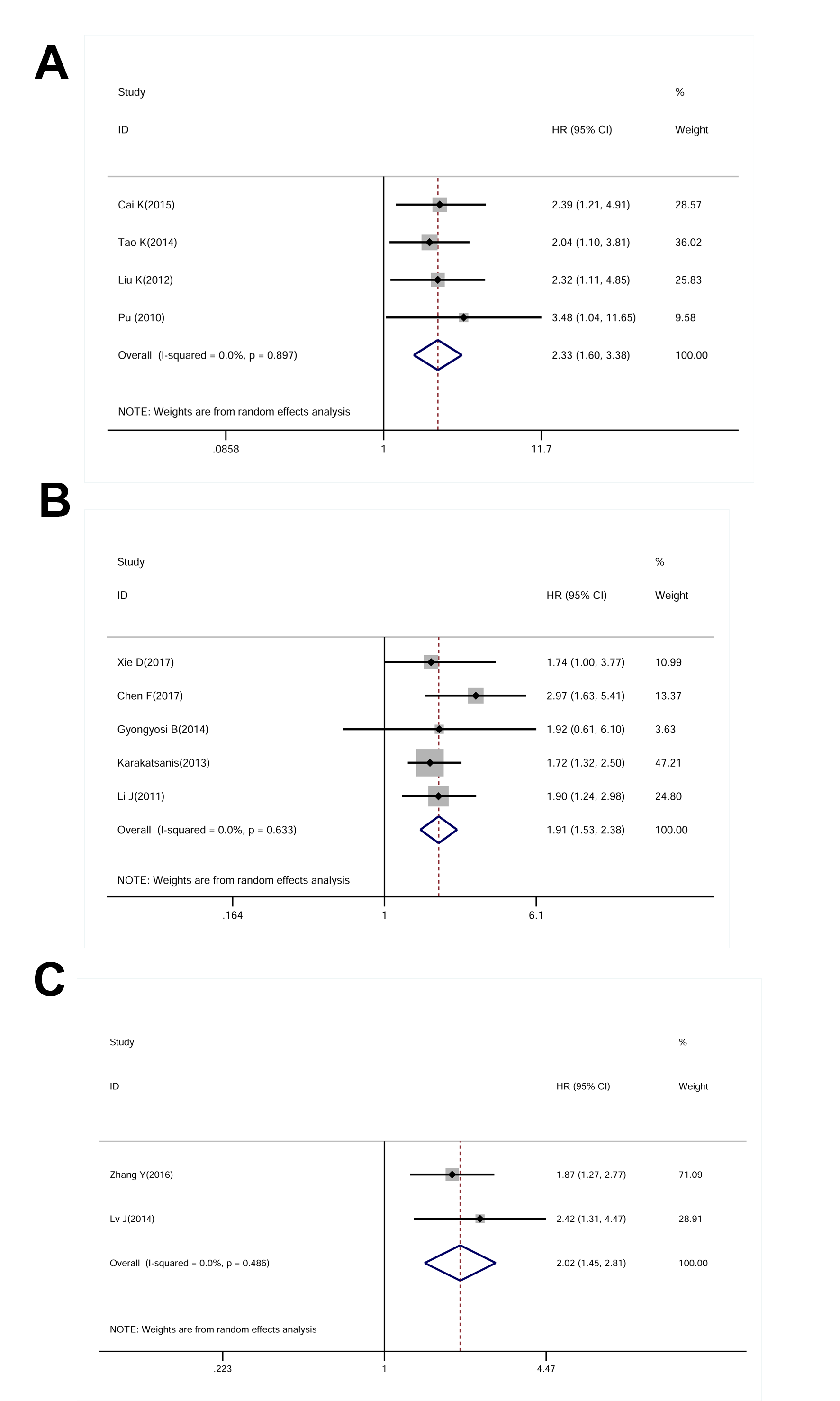

Supplement: Supplementary file 3 — Figure S3. Subgroup analyses of the studies that evaluated the HRs of high miR-221 expression as compared to low expression in OS by cancers, (A) Colon cancer (B) Live cancer (C) Lung cancer (TIF 15382 kb) [file 12885_2019_6079_MOESM3_ESM.tif]

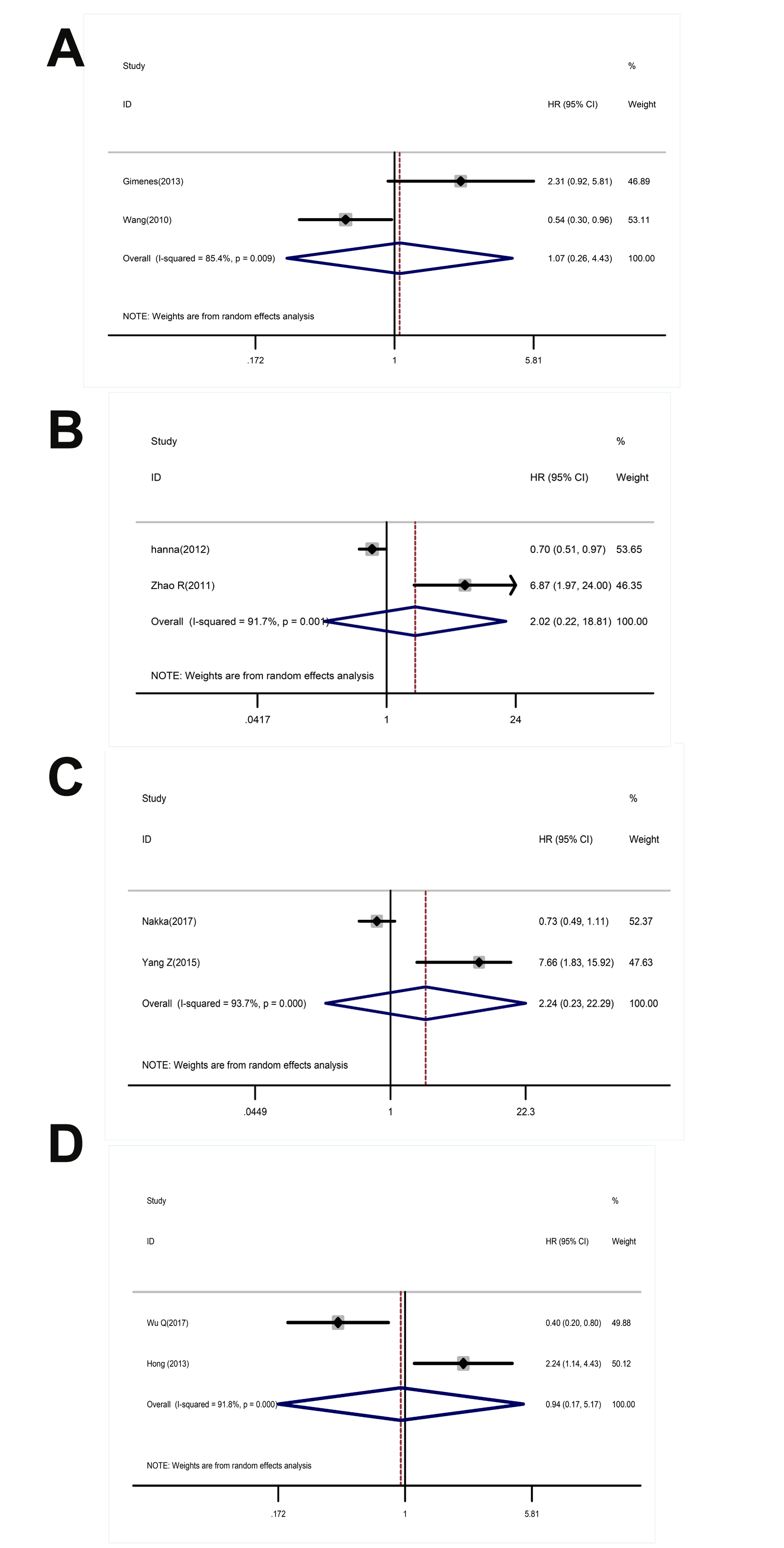

Supplement: Supplementary file 4 — Figure S4. Subgroup analyses of the studies that evaluated the HRs of high miR-221 expression as compared to low expression in OS by cancers, (A) ALL (B) Breast cancer (C) Osteosarcoma (D) Ovarian cancer (DOCX 19 kb) (TIF 14956 kb) [file 12885_2019_6079_MOESM4_ESM.tif]

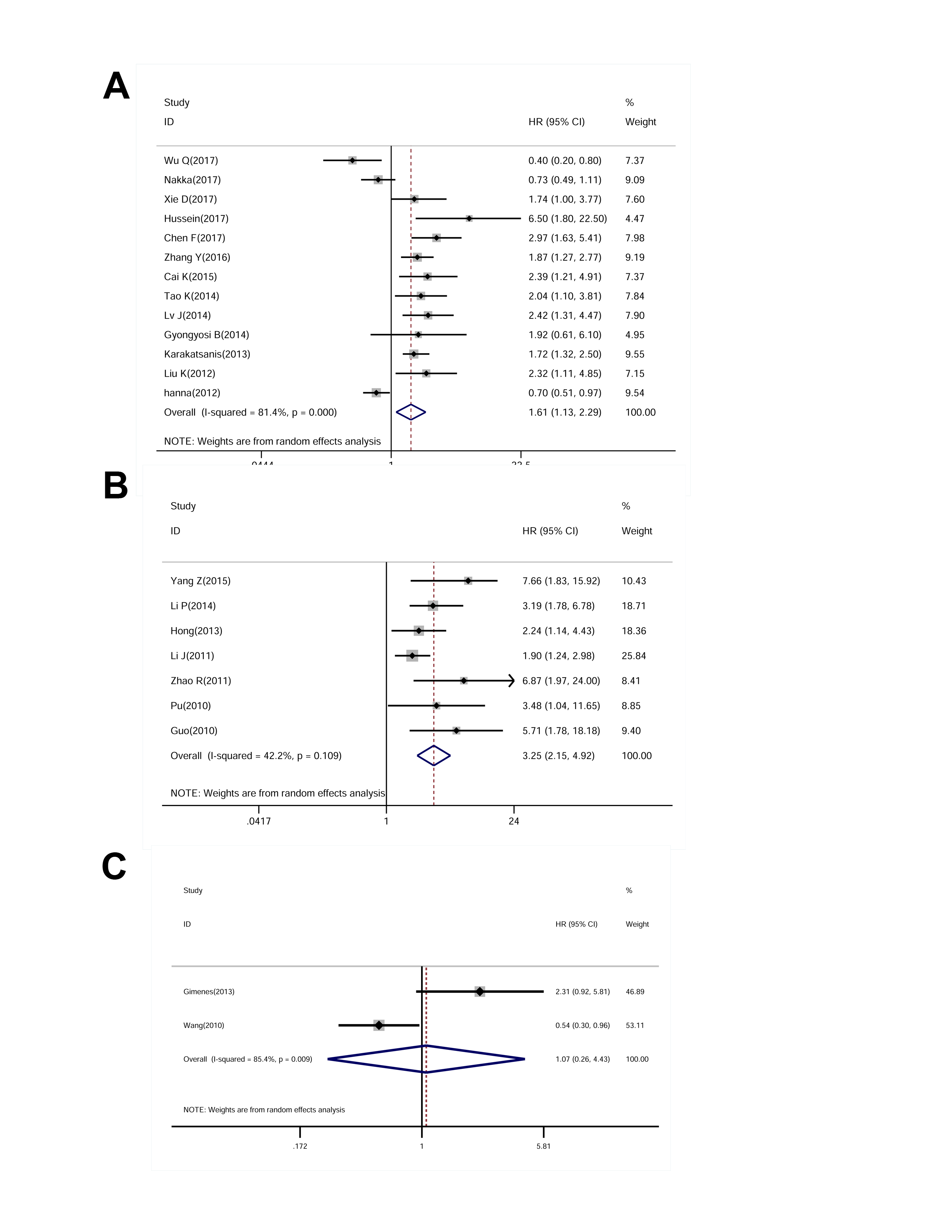

Supplement: Supplementary file 5 — Figure S5. Subgroup analyses of the studies that evaluated the HRs of high miR-221 expression as compared to low expression in OS by origins, (A) Tumor tissues (B) Serum/plasm (C) Marrow (TIF 24676 kb) [file 12885_2019_6079_MOESM5_ESM.tif]
