# Supplementary material for: Prognostic value of miR-221 in human malignancy: evidence from 3041 subjects
Source: BMC Cancer. 2019 Aug 30;19:867. doi: 10.1186/s12885-019-6079-1 (PMC6717359; doi:10.1186/s12885-019-6079-1)
Supplement: Supplementary file 6 — Table S1. Literature search strategy of PubMed database. The Embase database is searched in a similar way to PubMed. (DOCX 19 kb) [file 12885_2019_6079_MOESM6_ESM.docx]

**Literature search strategy**

| Note | Contents |
| --- | --- |
| #1 | “MIRN221 microRNA” [Mesh terms] |
| #2 | “MicroRNA 221” OR “MicroRNA-221” [Keywords] |
| #3 | “MiR 221” OR “MiR-221” [Keywords] |
| #4 | “Micro RNA 221” OR “Micro RNA-221” [Keywords] |
| #5 | “MiRNA 221” OR “MiRNA-221” [Keywords] |
| #6 | “MicroRNAs 221” [Keywords] |
| #7 | “MiRNAs 221” [Keywords] |
| #8 | “Micro RNAs 221” [Keywords] |
| #9 | #1 OR #2 OR #3 OR #4 OR #5 OR #6 OR #7 OR #8 |
| #10 | “Neoplasms” [Mesh terms] |
| #11 | “Lymphoma” OR “Sarcoma” OR “melanoma” [Mesh terms] |
| #12 | “leukemia” [Mesh terms] |
| #13 | “Tumor” OR “Tumors” [Keywords] |
| #14 | “Cancer” OR “Cancers” [Keywords] |
| #15 | “Carcinoma” OR “Carcinomas” [Keywords] |
| #16 | “Neoplasia” OR “Neoplasias” [Keywords] |
| #17 | “Malignancy” OR “Malignancies” [Keywords] |
| #18 | “Neoplasm” OR “Neoplasms” [Keywords] |
| #19 | “Lymphoma” OR “Lymphomas” [Keywords] |
| #20 | “Sarcoma” OR “Sarcomas” [Keywords] |
| #21 | “leukemia” OR “leukemias” [Keywords] |
| #22 | “melanoma” OR “melanomas” [Keywords] |
| #23 | #10 OR #11 OR #12 OR #13 OR #14 OR #15 OR #16 OR #17 OR #18 OR #19 OR #20 OR # 21 OR #22 |
| #22 | #9 AND #23 |

**Table S1**: Literature search strategy of PubMed database. The Embase database is searched in a similar way to PubMed.
